# The importance of planning for a seamless transition in an adaptive phase II randomised trial

**DOI:** 10.1186/1745-6215-12-S1-A7

**Published:** 2011-12-13

**Authors:** Andrea Marshall, Janet A Dunn, Julie Fletcher, Helen B Higgins, Christopher J Poole

**Affiliations:** 1 Warwick Clinical Trials Unit, University of Warwick, Coventry, West Midlands, CV4 7AL, UK

## Objectives

To investigate the importance of incorporating adaptive designs of randomised phase II trials into the protocol and patient information sheets using NEO-ESCAPE as an illustrative example.

## Methods

NEO-ESCAPE was a randomised two arm phase II study in inoperable ovarian cancer designed as an external pilot to inform a future phase III randomised controlled trial. The primary objective was to assess the feasibility of two new extended chemotherapy regimens with 44 patients required on each arm. If one or both treatment arms proved feasible, the trial would continue to recruit to the feasible treatment arm(s) to improve the estimates of outcomes to be used in the phase III trial. Stopping rules were essential to enable pre-planned decisions on the futility of continuing to the required 44 patients on each arm. Simulations to assess futility were used in the decision making process.

## Results

At the first pre-planned interim analysis when 56 patients had been recruited (28 on each arm) and 21 patients (11 on Arm A; 10 on Arm B) had finished treatment, it was clear that one arm of the trial would not meet the feasibility criteria on completion of recruitment. On the advice of the Independent Data Monitoring Committee, this arm of the study closed to recruitment but follow-up for these patients continued.

Closure of one arm of the study resulted in a temporary halt of the trial while the protocol was amended to a single arm study, the patient information sheet was updated and all the relevant ethics, MHRA and Research and Development approvals were obtained. This temporary two month halt in recruitment to the study carried not only a loss of potential patients during the closed period but also a loss of momentum of centres on re-opening of the study and hence resulted in a delay in completing recruitment.

## Conclusions

Our experiences with this phase II study emphasise the need for full details of any adaptive designs, including all anticipated scenarios and subsequent actions, to be fully incorporated into the protocol and patient information sheet to avoid the need for a temporary halt of recruitment. A detailed statistical analysis plan is also essential in ensuring optimal decision making within these types of studies. The dilemma remains on how best to achieve a seamless transition in the adaptive element whilst not confusing patients with too much information about each possible future adaptation.

